# Sotrovimab use in Japanese inpatients with COVID-19: post-infusion adverse events

**DOI:** 10.1186/s12879-022-07889-z

**Published:** 2022-12-03

**Authors:** Junichi Yoshida, Kenichiro Shiraishi, Masao Tanaka

**Affiliations:** 1 Infection Control Committee, Shimonoseki City Hospital, 1-13-1 Koyo-Cho, Shimonoseki, 750-8520 Japan; 2 Department of Medicine and Biosystemic Science, Kyushu University Faculty of Medicine, 3-1-1 Maidashi, Higashi-Ku, Fukuoka, 812-8582 Japan

**Keywords:** SARS-CoV-2, Sotrovimab, Fever, Hypoxia, Infusion reaction

## Abstract

**Background:**

Sotrovimab neutralizing SARS-CoV-2 remained effective at the advent of B.1 lineage of the Omicron variant in outpatients. Primarily for hospitalized patients, however, the Japanese government regulated to administer this antibody agent. As this regulation enabled close monitoring in inpatients to investigate post-infusion adverse events (AEs) and efficacy, we attempted a retrospective study while the Omicron BA.1 lineage was dominant regionally.

**Methods:**

Subjects were inpatients with COVID-19 who received infusion of sotrovimab in our institute. In line with previous clinical trials, we included patients at risk of COVID-19 worsening and SARS-CoV-2 vaccinees, who were hospitalized as directed by the government. For statistical analyses, we reviewed background factors of demographics, imaging, and laboratory findings for the outcome infusion-related reactions including post-infusion pyrexia over 38 degrees Celsius and/or pulse oximetry below 94%.

**Results:**

In a total of 139 patients, the follow-up period had a median of 200 days (range, 154–248 days). Among 119 patients (85.6%) fully vaccinated for SARS-CoV-2, 86 (61.9% of all) underwent 2 doses while 33 (23.7% of all) received 3 doses. For the outcome of pyrexia and/or dyspnea (N = 40, 28.8%), multivariate analysis showed that significant risk factors were pre-infusion lowered oximetry below 96.5% (Odds Ratio [OR] 0.344, 95% Confidence Interval [CI] 0.139–0.851, P = 0.021) and pre-infusion temperature more than 36.7 degrees Celsius (OR 4.056, 95% CI 1.696–9.701, P = 0.002). Infusion-related reactions included vomiting immediately after infusion, chill/shivering, dizziness, rash, pruritus, pyrexia, and dyspnea. The number of patients with any of these events was 44 (31.6%). Three patients (2.2%) showed worsening of COVID-19; one developed hypoxia and two died. Limitations for this study included no genome typing whether BA.1 or BA.2 lineage of the Omicron variant but the local epidemiology indicated the prevalence of BA.1. Another was sotrovimab administration for inpatients that allow precise detection of post-infusion events, confounding previous exacerbation definition including hospitalization.

**Conclusions:**

For 24 h after infusion of sotrovimab, COVID-19 patients showing pre-infusion lowered oximetry below 96.5% and/or temperature more than 36.7 degrees Celsius may have temperature elevation or dyspnea, warranting close monitoring for these risk factors.

## Background

Sotrovimab reportedly has its roots in an individual who had recovered from the 2003 outbreak of severe acute respiratory syndrome (SARS) [1] and it binds to a cryptic receptor binding domain epitope [2].

Thus, sotrovimab has remained as one of a few antibody drugs neutralizing activity against the Omicron pseudovirus [2] while the Omicron variant escapes various neutralizing antibodies [3]. The clinical trials in COVID-19 outpatients showed its adverse effects (AE) including fever and hypoxia for 24 h as “infusion-related reaction” [4]. From the US and Europe, however, a study group [5] described that sotrovimab was not efficacious for the hospitalized patients with symptoms for up to 12 days. For inpatients, however, the Japanese administration recommended the use of sotrovimab within the limit of COVID-19 symptoms for up to 5 days [6].

To analyze factors to predict post-infusion AEs of COVID-19 for inpatients to prepare for close monitoring, we retrospectively investigated post-infusion AEs, and their risk factors of sotrovimab.

## Methods

Subjects were a consecutive series of inpatients positive for antigen or polymerase chain reaction of SARS-CoV-2, who underwent intravenous administration of sotrovimab 500 mg (Xevudy; GlaxoSmithKline, Tokyo, Japan) in a single institute. The entry criteria were mostly in accordance with previous clinical trials [4], which excluded SARS-CoV-2 vaccinees. The Japanese regulatory authority [6], however, permits previous vaccination in the domestic criteria. The Clinical Trial described an entry criterion of body mass index (BMI) more than 30 kg/m^2^ [4], but we observed another level of more than 25 kg/m^2^ by US Food and Drug Administration [7]. Otherwise, patients were included for an onset of COVID-19 symptoms within the previous 5 days and those with risk factors for progression of COVID-19 such as older age (≥ 55 years), diabetes mellitus, chronic kidney disease, congestive heart failure, chronic obstructive pulmonary disease, or bronchial asthma [4]. We added connective tissue diseases, hematologic diseases, hepatic disease, cancer, or smoking for inclusion criteria.

The exclusion criteria were patients with pulse oximetry below 94% or in need of supplemental oxygen [4]. We excluded patients with computed tomography (CT) showing ground glass attenuation in the bilateral lungs, because it runs risk of transient decrease in pulse oximetry demonstrated in our previous investigation using casirivimab/imdevimab [8]. Also, we excluded patients showing body temperature of 38.0 degrees Celsius or more at presentation because post-infusion fever was difficult to distinguish between AE and the course of the disease.

Methods were retrospective review of background factors influencing the outcome of 24-h post-infusion obtained from the electronic health record. Patients received supplemental oxygen at a pulse oximetry of 94% or below.

The background factors included (1) demographics such as age, gender and BMI, (2) smoking status, (3) symptomatic days, or (Infusion Day)—(Onset Day), (4) body temperatures and pulse oximetry before infusion, (5) pre-infusion hematological and biochemical results, and (6) the doses of SARS-CoV-2 vaccination.

We defined the infusion-related reactions as was in the clinical trial [4], including pyrexia, chills, dizziness, dyspnea, pruritus, rash, and other anaphylactoid reactions within 24 h after administration of sotrovimab. Of them, the pyrexia was defined as post-infusion body temperature elevation above 38.0 degrees Celsius or Grade 1 AE according to the Common Terminology Criteria for Adverse Events (CTCAE) [9]. We specified the duration as 24-h based on the clinical trial [4]. Secondly, we defined dyspnea as pulse oximetry decrease below 94%.

We also investigated biochemical abnormality, worsening of COVID-19 such as delayed hypoxia in need of oxygen supply or re-admission, and all cause death within 29 days after administration of sotrovimab. Patients were discharged in accordance with the domestic regulations [6].

Statistical analyses designated that the major outcome as pyrexia and/or dyspnea in infusion-related reactions and that 2 or 3-dose vaccinees were regarded as fully vaccinated while those unknown for vaccination history were treated as no vaccinees. The analyses included receiver-operating characteristics (ROC) analysis for continuous variables to determine cutoff values.

Variables more than the cutoff were given a value of 1, but that of age below the cutoff was given a value of 1. In the logistic regression analysis using the stepwise method on the software SPSS Version 26 (IBM Japan, Tokyo, Japan), variables with P values of 0.05 or less at univariate analysis underwent subsequent multivariate analysis. Statistical significance was defined as P less than 0.05.

## Results

Sotrovimab was given from 27 December 2021 through 31 March 2022, when the BA.1 lineage of the Omicron variant of SARS-CoV-2 was dominant and BA.2 lineage accounted for 0.5% in the country [10]. Of a total of 149 inpatients subjected to the current study, 139 patients were analyzed because remaining 10 patients had a body temperature of 38.0 degrees Celsius at presentation. The follow-up period was a median of 200 days (range, 154–248 days). Their median age was 69 years while men accounted for 73 patients and women for 66 patients (Table 1).

**Table 1:** Profile of background factors by temperature increase and oximetry decrease 24-h post-infusion of sotrovimab and the area under curve (AUC) of the receiver operating characteristic analysis for the outcome of fever and/or dyspnea

Factor	Division (Unit)	Total		Receiver operating characteristics analysis			
		N = 139	Median (Inter-quartile range)	AUC	95% CI, Lower	95% CI, Upper	P value
Sex	Male	73					0.999
	Female	66					
Smoking	Yes	65					0.627
	Never	74					
2 or 3-Dose Vaccination	Yes	119					0.687
	No	20					
Age	(Years)		69.0 (54.0–85.0)	0.49	0.385	0.595	0.831
Body Mass Index	(kg/m^2^)		22.4 (19.9–25.9)	0.425	0.313	0.537	0.017
Symptomatic	(Day)		2.0 (1.0–3.0)	0.399	0.301	0.497	0.022
PreTemp	Celsius		36.7 (36.2–37.0)	0.765	0.679	0.852	< 0.001
PreOx	(%)		97.0 (96.0–98.0)	0.36	0.251	0.468	0.002
While Blood Cell	(× 10^3^)		5.05 (4.04–6.02)	0.585	0.472	0.698	0.172
Neutrophil	(%)		62.6 (53.5–70.7)	0.693	0.594	0.791	0.007
Lymphocytes	(%)		23.9 (19.0–32.5)	0.287	0.195	0.38	0.001
C-reactive protein	(mg/dl)		1.11 (0.36–2.59)	0.603	0.501	0.706	0.136
Total Bilirubin	(mg/dl)		0.50 (0.40–0.70)	0.532	0.426	0.638	0.699
Creatinine	(mg/dl)		0.84 (0.68–1.11)	0.619	0.512	0.726	0.145
Platelet	(× 10^3^)		182.0 (143.3–224.8)	0.392	0.288	0.497	0.077

PreTemp, pre-infusion body temperature more than 36.7 degrees Celsius; PreOx, pre-infusion pulse oximetry below 94%; CI, confidence interval

The risk factors for the administration included elderly age (N = 105, 75.5%), high BMI (N = 4, 2.9%), connective tissue diseases (N = 2, 1.4%), cardiac diseases (N = 11), diabetes mellitus (N = 2, 1.4%), hematologic diseases (N = 2, 1.4%), hepatic disease (N = 1, 0.7%), cancer (N = 1, 0.7%), pulmonary diseases (N = 10, 7.2%), renal diseases (N = 3, 2.2%), and smoking (N = 9, 6.5%). Two patients (1.4%) receiving oxygen supply, however, underwent sotrovimab administration as failures of the exclusion criteria [4].

Among 119 fully vaccinated patients (85.6%) for SARS-CoV-2, 2-dose recipients accounted for 86 (61.9% of all) while 3-dose vaccinees were 33 (23.7% of all) patients.

Infusion-related reactions included vomiting immediately after infusion, chill/shivering, dizziness, rash, pruritus, pyrexia, and dyspnea but no anaphylactoid reactions (Table 2). The number of patients with any of these events was 44 (31.6%). The median increase of body temperature from the pre-infusion level was 0.9 (range, − 0.3–3.1) degrees Celsius.

**Table 2:** Number of patients developing adverse event of infusion-related reaction 24 h after administration of sotrovimab

	Patients	(N = 139)
Adverse events		(%)
Chill/shivering	5	3.6
Vomiting	1	0.7
Dizziness	1	0.7
Rash	3	2.2
Pruritus	2	1.4
(A) Fever > 38.0 Celsius	33	23.7
(B) Dyspnea < 94% oximetry	15	10.8
(A) and/or (B)	40	28.8
Patients with any events	44	31.7

Of a total of 149 patients undergoing sotrovimab, 139 patients were subjected because remaining 10 patients had a pre-infusion temperature of 38.0 degrees Celsius

To undergo logistic regression analysis, continuous variables were tested for the area under curve of ROC analysis and were determined for cutoff values (Table 1) for the following analysis. For example, the cutoff value of pyrexia was 36.7 degrees Celsius and that of pulse oximetry was 96.5%.

**Fig. 1:**
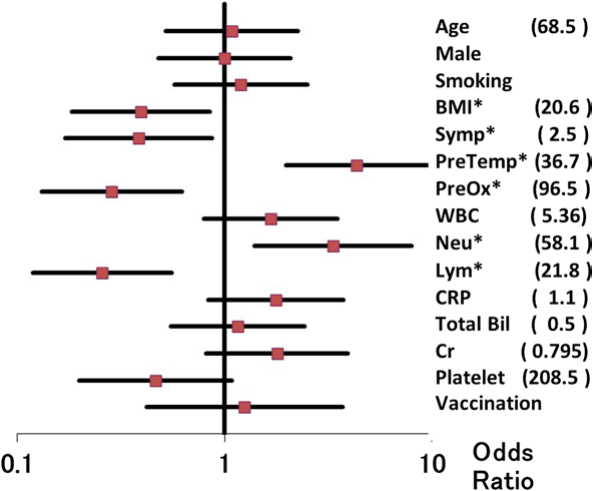
Forest plots of the univariate analysis showing risk factors for (1-a) temperature increase over 38 degrees Celsius and (1-b) decrease in pulse oximetry below 94% for 24-h after sotrovimab infusion. Bar, 95% confidence interval; dot, odds ratio; BMI, body mass index (kg/m^2^); Sympt, symptomatic days; PreTemp, pre-infusion body temperature more than 36.7 degrees Celsius; PreOx, pre-infusion oximetry below 94%; WBC, white blood cell (× 10^3^); Neu, neutrophils (%); Lym, lymphocytes (%); CRP, C-reactive protein (mg/dl); Bil, bilirubin (mg/dl); Vaccination, vaccinee of 2- or 3-dose; Cr, creatinine (mg/dl); Platelet (× 10^3^); *statistical significance; (), cutoff values determined at the receiver operating characteristics analysis

For the infusion-related reaction of pyrexia and/or dyspnea (N = 40, 28.8%), the univariate analysis showed 6 factors with statistical significance (Fig. 1), including pre-infusion temperature and oximetry, BMI, symptomatic days, the percentages of neutrophiles and lymphocytes. Among these, the multivariate analysis showed that significant risk factors were pre-infusion lowered oximetry (Odds Ratio [OR] 0.344, 95% Confidence Interval [CI] 0.139–0.851, P = 0.021) and pre-infusion temperature more than 36.7 degrees Celsius (OR 4.056, 95% CI 1.696–9.701, P = 0.002). Additionally, 8 (5.8%) showed temperature increase over 39.0 degrees Celsius. Patients developing dyspnea received oxygen supplementation for a median of 1.5 days (range, 1–5).

During hospitalization, we observed elevated aminotransferase levels (N = 1, 0.7%). A total of three patients (2.2%) showed worsening of COVID-19. In detail, one (0.7% of all) showed hypoxia leading into re-admission and two (1.4% of all) died as follow. A 90-years-old female developed thrombus in the left atrium on 6th post-infusion day to Infectious Disease Ward as worsening after temporary recovery with demise three days later. The other, a 96-years-old woman having had compromised renal function at infusion became re-admitted on post-infusion day 13 and died on the following day with acute renal failure. If or not sotrovimab influenced the death remained uncertain.

## Discussion

Our study revealed that 24-h after infusion of sotrovimab, pyrexia and/or dyspnea were related to pre-infusion body temperature > 36.7 degrees Celsius and oximetry below 96.5%. These findings warrant close monitoring for patients showing these risk factors.

As regards (1) pyrexia, 73 of 430 patients in the sotrovimab group (17%) reported AE, in which 6 (1%) showed any infusion-related reaction in the clinical trial [4]. Similarly, Gupta et al. [4] reported that in COVID-19 patients ≤ 5 days after the onset of symptoms, any infusion-related reactions were noted in 1% in sotrovimab group (N = 136). Their protocol, however, stated that infusion-related reactions were documented as Grade 2 (> 39.0 degrees Celsius) or more, which in our series corresponds to a rate of 5.8%.

For (2) post-infusion lowered oximetry, Gupta and others [11] also defined pulse oximetry below 92% or oxygen supplementation as infusion-related reaction. We defined, however, oximetry below 94% as decrease in oximetry as was defined for an exclusion criterion in the clinical trial [4] and we observed a hypoxia rate of 10.8%. Another reason for the discrepancy may owe to close in-hospital monitoring rather than in outpatients as in previous studies.

These events, the temperature increase and oximetry decrease, may reflect antibody-dependent enhancement (ADE) [12] and enhanced respiratory disease [13], respectively. For pyrexia, an independent risk factor was young population prone to produce antibody after vaccination [14]. The logistic regression analysis in our study, however, failed to show association between the previous vaccination and fever and/or dyspnea (Fig. 1), thus the issue of ADE remains to be investigated. For reduced oximetry, delayed infusion may have produced antibody when the antibody drug, sotrovimab, may have brought about enhanced respiratory distress [13].

As for the efficacy of sotrovimab, Ong et al. [15] described that it was efficacious for patients with early administration and at risk of COVID-19 worsening in their real-world use. Likewise, Kow and others [16] showed the importance of early administration of neutralizing antibody agents to prevent ultimately death from COVID-19. We experienced a demise by thrombus formation in the left atrium, which we presumed as COVID-19 exacerbation having little to do with sotrovimab administration. A literature review on similar cases after administration of sotrovimab revealed an anecdotal report of developing aortic arch thrombus on post-infusion Day 10 [17].

A limitation for this study was the lack of viral lineage analysis in our region but the local epidemiology indicated the prevalence of BA.1 lineage. As of this writing for revision at the time of BA.5 prevalence, neutralizing efficacy of sotrovimab became lowered but the message of our study may apply for future neutralizing antibody agents. Rockett et al. [18] reported that sotrovimab was efficacious for SARS-CoV-2 Delta variant but sotrovimab may have partially worked as in the report describing in vitro activity for BA.1 but limited activity for BA.2 lineage [19]. Furthermore, Rockett et al. [18] demonstrated that sotrovimab use may invite resistance mutations in the Delta variant. These considerations, however, await future investigation because the current study rested on the assumption that the BA.1 lineage had been prevalent throughout our study.

## Conclusions

For 24 h after infusion of sotrovimab, COVID-19 patients showing pre-infusion lowered oximetry below 96.5% and/or temperature more than 36.7 degrees Celsius may have temperature elevation and dyspnea, warranting close monitoring for these risk factors.

## Data Availability

The datasets used and/or analyzed during the current study are available from the corresponding author on reasonable request.

## Abbreviations

SARS: Severe acute respiratory syndrome
AE: Adverse effects
BMI: Body mass index
CT: Computed tomography
ROC: Receiver-operating characteristics
OR: Odds ratio
CI: 95% Confidence interval
IL: Interleukin
ADE: Antibody-dependent enhancement
